# Amyand’s Hernia and the Desarda Repair: A Case Series and Contemporary Review of Management Strategies

**DOI:** 10.7759/cureus.94468

**Published:** 2025-10-13

**Authors:** Tania Gupta, Sarah Cook, Sonia Lele, Alexander Simmonds, Patrick D Melmer

**Affiliations:** 1 Surgery, Virginia Commonwealth University School of Medicine, Richmond, USA; 2 Surgery, Northwestern University Feinberg School of Medicine, Chicago, USA; 3 Surgery, University of Southern California Keck School of Medicine, Los Angeles, USA; 4 Surgery, Virginia Commonwealth University, Richmond, USA

**Keywords:** acute care surgery and trauma, amyand’s hernia, groin hernia, mesh repair, surgical acute abdomen

## Abstract

Treatment guidelines regarding appendix-containing groin hernias lack clarity with respect to tissue versus mesh repair in cases that are considered clean-contaminated following appendectomy. We review the management of Amyand’s hernia based on currently available literature and describe the cases of three adult male patients who underwent operative repair of Amyand’s hernias. Case 1 involves a 64-year-old man who underwent Desarda tissue repair and appendectomy for intraoperatively discovered appendicitis. Case 2 involves a 48-year-old man who underwent appendectomy and Desarda tissue repair due to an ischemic appendix. Case 3 involves a 71-year-old man with a complex cardiac history who underwent open Lichtenstein repair with absorbable biosynthetic mesh following ileocecectomy for cecal ischemia. These cases are discussed in the context of current management guidelines and published literature regarding tissue repair versus mesh placement and the role of appendectomy. Most available literature supports appendectomy and tissue repair in cases of appendicitis or contamination. However, individualized patient factors, including anatomy, contamination risk, and recurrence risk, should guide the choice of repair technique.

## Introduction

Amyand’s hernia is a rare variant of groin hernia in which the vermiform appendix is contained within the hernia sac, accounting for approximately 1% of all inguinal hernias [1]. In pediatric patients, it is typically due to persistence of the peritoneo-vaginal canal, whereas in adults, fibrotic attachments between the appendix and hernia sac may guide herniation [1]. Contraction of the abdominal wall can cause luminal obstruction and subsequent appendicitis. Most patients with Amyand’s hernia present emergently with bowel obstruction, and the diagnosis is often made intraoperatively [2]. This can complicate treatment as the discovery of Amyand’s hernia may necessitate unanticipated changes to the preoperative treatment plan. Because of its rarity, consensus on management is limited, particularly regarding whether to perform appendectomy when the appendix is normal and whether to use mesh versus tissue-based repairs in potentially contaminated fields. Mesh repair (e.g., Lichtenstein technique) utilizes a synthetic or biosynthetic mesh for reinforcement, whereas tension-free tissue repairs avoid the use of prosthetic material in favor of native tissue. One such technique, the Desarda method, utilizes an undetached strip of external oblique aponeurosis to reinforce and provide functional support for the posterior wall of the inguinal canal during muscular contraction.

We aim to add to the literature regarding this interesting entity and the varying approaches based on the operative situation through a discussion of surgical management of three patients with Amyand’s hernias treated at a single urban, quaternary academic institution in the context of currently available literature.

## Case presentation

Case 1

**Figure 1:**
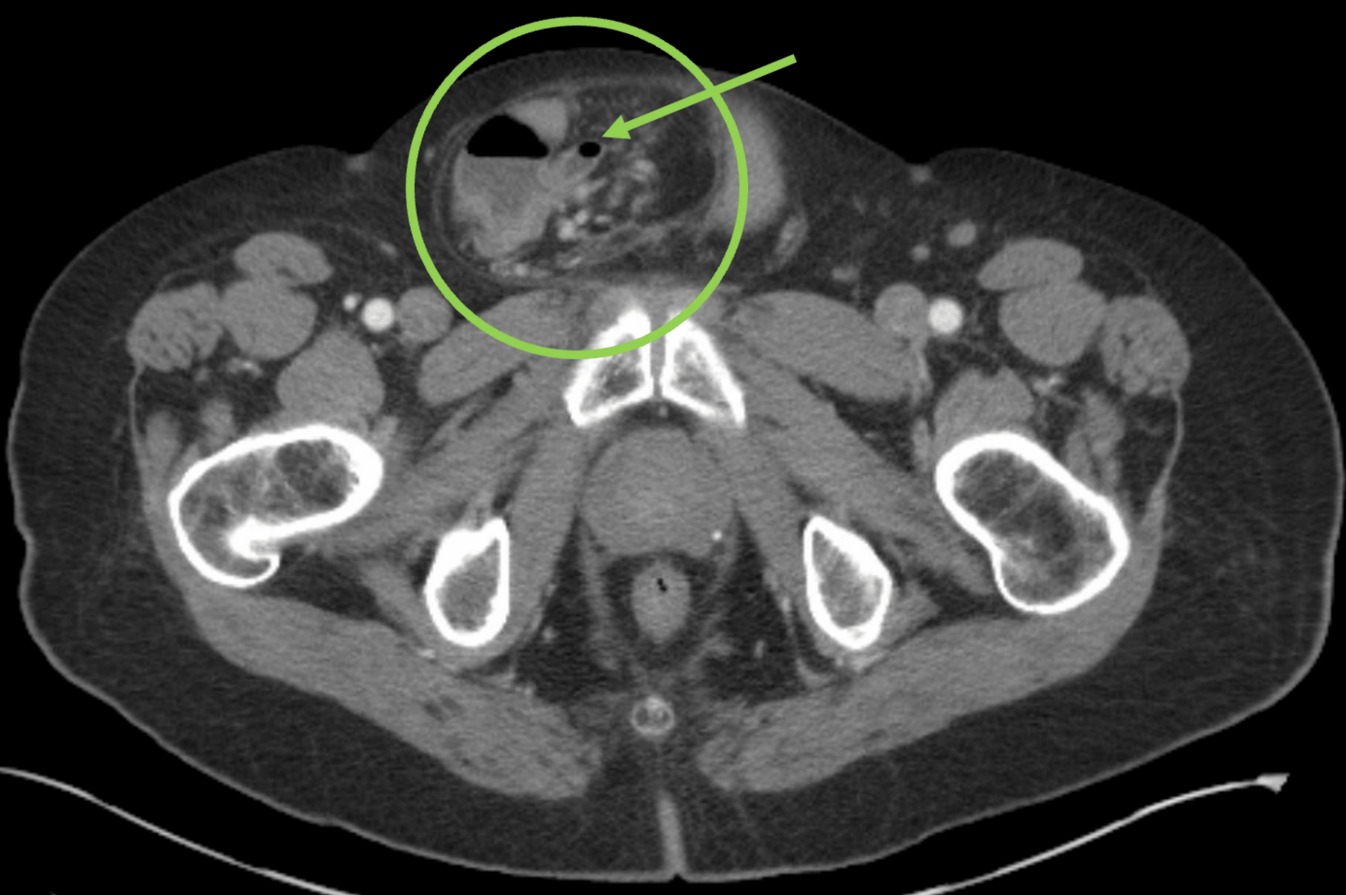
Amyand's hernia with bowel in the scrotum (green circle), with an inflamed appendix (green arrow) noted.

A previously healthy 64-year-old male presented to the emergency department (ED) with two days of nausea, vomiting, and non-localizing, dull abdominal pain. History was significant for venous insufficiency and a large bowel-containing right inguinal hernia diagnosed six years prior. He endorsed having bowel movements and passing flatus. Physical exam revealed a large, reducible, right inguinoscrotal hernia without abdominal tenderness or guarding. Laboratory results were significant for elevated lactate and creatinine with no leukocytosis. Abdominal computed tomography (CT) demonstrated his known large right-sided inguinal hernia containing the cecum, appendix, and terminal ileum, as well as dilated central small bowel with air fluid levels consistent with obstruction (Figure 1).

The patient’s elevated lactate and creatinine were attributed to dehydration associated with recent intolerance to oral intake (Table 1).

**Table 1:** Laboratory values and reference ranges

Parameter	Patient Value	Reference Range
Lactate	2.1 mmol/L	0.5–2.0 mmol/L
WBC	15.5 ×10⁹/L	3.7–9.7 ×10^9^/L
Creatinine	1.45 mg/dL	0.60–1.20 mg/dL

**Figure 2:**
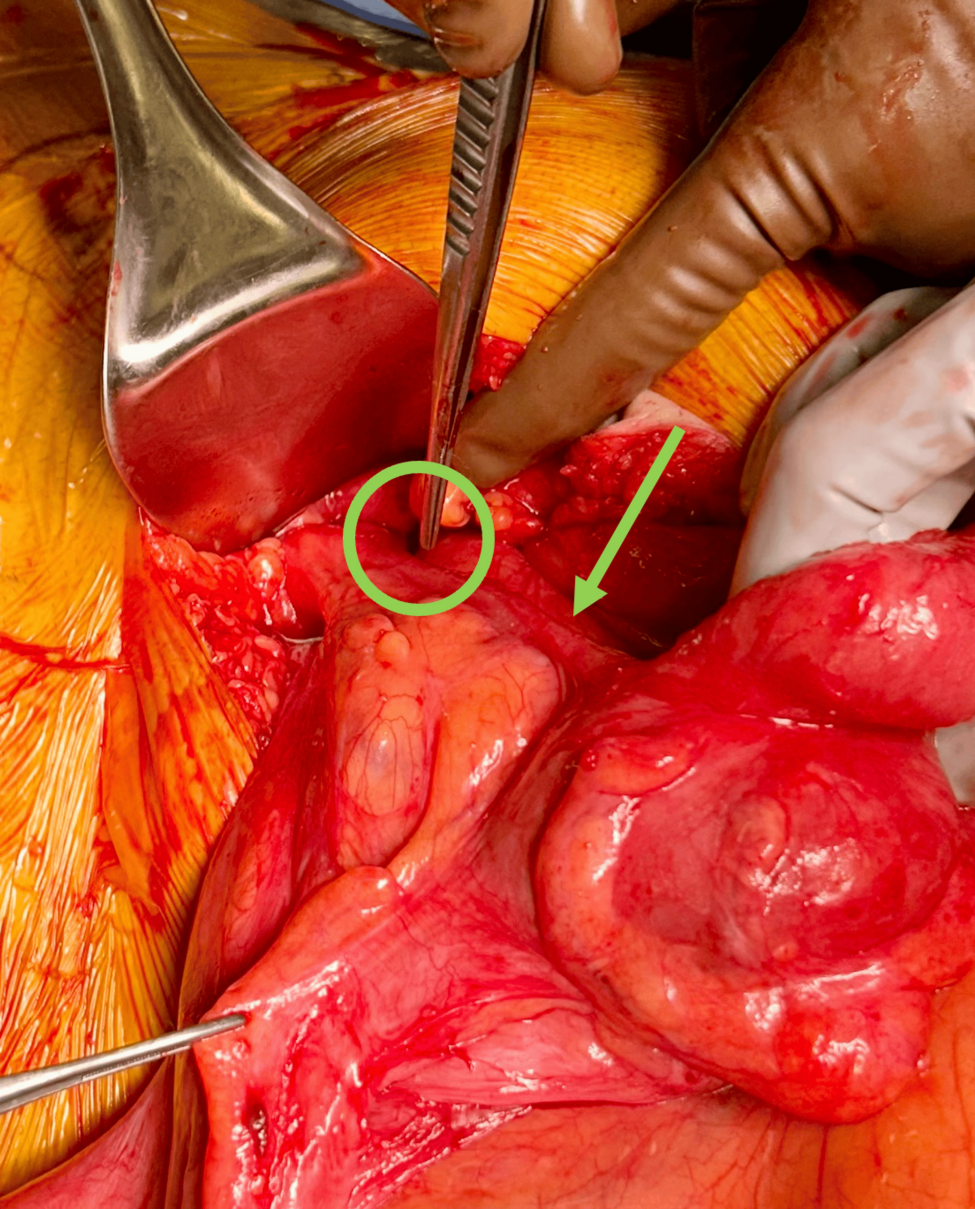
Amyand's hernia defect (green circle), with an inflamed appendix (green arrow) reduced from the groin.

He was admitted to the emergency general surgery service for a trial of nonoperative management but had worsening abdominal pain over the ensuing 24 hours. Due to the failure of non-operative management, the decision for robotic herniorrhaphy was made with the patient’s consent. During the operation, the case was converted to an open approach due to the presence of bowel with ischemic change (Figure 2). The small bowel was reduced from the groin and was deemed to be viable. However, appendicitis was noted, and an appendectomy was performed. The hernia was repaired without mesh via the tissue-based Desarda technique. The patient had an uneventful recovery, and no recurrence was noted at the three-month follow-up.

Case 2

**Figure 3:**
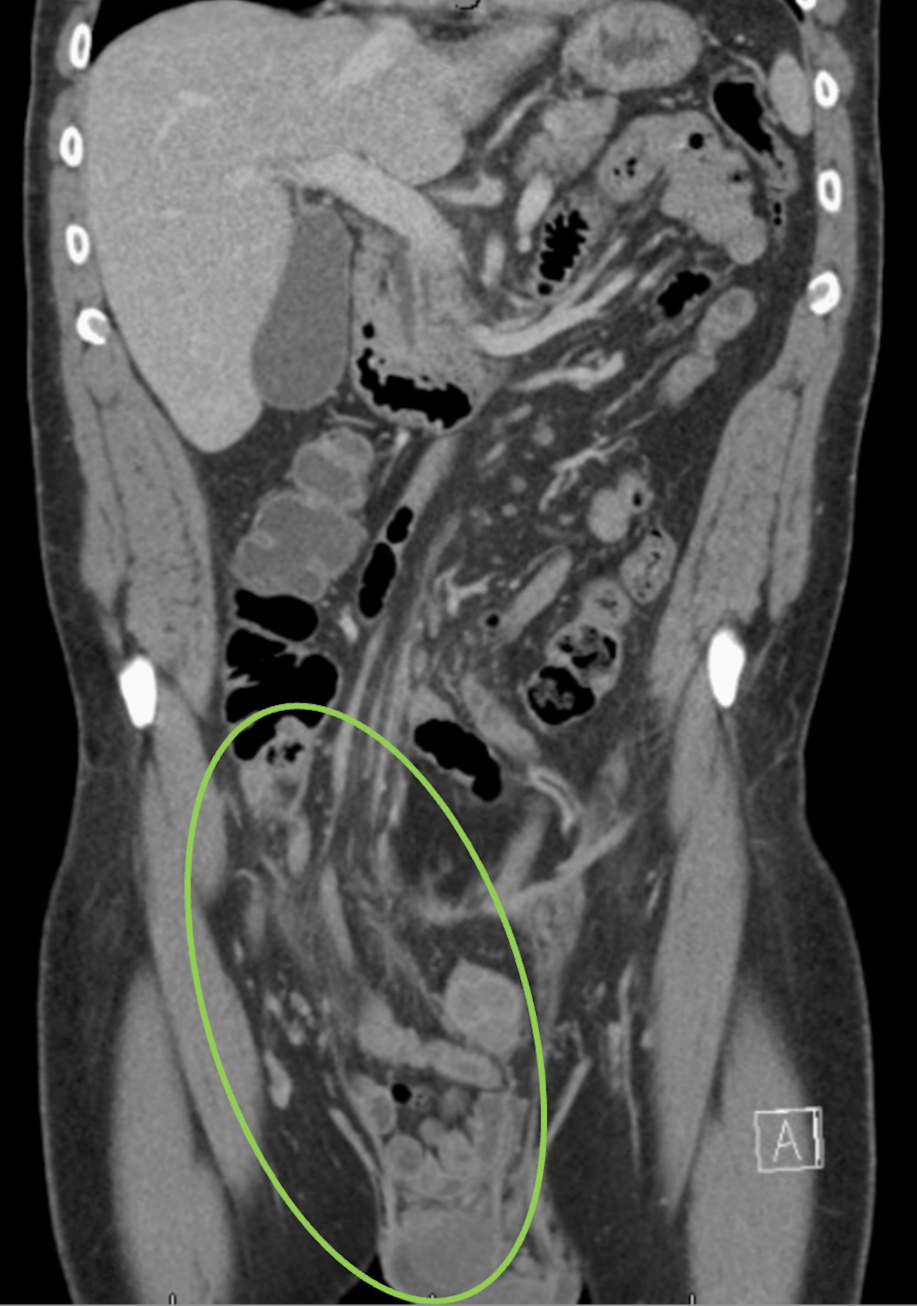
Inguinoscrotal hernia involving multiple distal small bowel loops, the appendix, and the proximal large bowel (green oval).

A 48-year-old male presented to the ED with one month of right-sided inguinal swelling and pain. History was significant for bilateral inguinal hernias status post open repair of an incarcerated left hernia repair with mesh placement six months prior. He endorsed nausea and vomiting but denied fevers, chills, or obstipation. CT demonstrated a large, right inguinoscrotal hernia involving multiple distal small bowel loops, the appendix, and the proximal large bowel (Figure 3).

He was taken emergently to the operating room (OR) for an open inguinal hernia repair. The bowel remained incarcerated despite opening the inguinal floor; therefore, laparotomy was performed to allow successful reduction. The mesoappendix was fused to the hernia sac, and the appendix was ischemic. Appendectomy was performed. To decrease the risk of future infection in this clean-contaminated case, the decision was made to conduct a primary tissue repair with the Desarda technique rather than a mesh placement. The patient had an uneventful recovery and no recurrence noted at the three-month follow-up.

Case 3

A 71-year-old male presented to the ED with acute right lower quadrant abdominal pain. Past medical history included heart failure with a left ventricular assist device in place and chronic anticoagulation use. He denied flatus and was exquisitely tender throughout the abdomen, right inguinal canal, and scrotum. Due to the concern for strangulated hernia, he was taken emergently to the OR. At laparotomy, he had cecal ischemia and underwent ileocecectomy with primary ileocolonic anastomosis and tension-free right inguinal hernia repair with resorbable poly-4-hydroxybutyrate biosynthetic mesh. The patient had a postoperative ileus that resolved with conservative management and otherwise recovered uneventfully. He had no recurrence at the three-month follow-up.

## Discussion

Amyand’s hernias have an estimated mortality rate of 14-30%, attributable primarily to sepsis, underscoring the importance of appropriate treatment [3]. However, due to the rare incidence of Amyand’s hernia, guidelines regarding specific treatment are unclear. In particular, the therapeutic approach to Amyand’s hernia repair has been controversial in terms of the use of mesh placement versus tissue repair for clean-contaminated cases and the necessity of appendectomy for a non-inflamed appendix within the hernia.

The Losanoff and Basson classification remains the most widely referenced framework, classifying cases into four types by the presence of appendicitis and other abdominal pathology [4]. These criteria have been successfully applied to patients with appendiceal hernias with good outcomes and generally recommend appendectomy in all cases of Amyand’s hernia, with mesh placement reserved for only cases of a normal appendix with no appendicitis [5-8].

In our series, all three patients had type 2 Amyand’s hernias - acute appendicitis within an inguinal hernia without diffuse peritonitis - and were treated with appendectomy. This aligns with existing recommendations for appendectomy in the presence of inflammation. While the Losanoff and Basson criteria recommend appendectomy in all cases of Amyand’s hernia, there have also been documented cases of patients without appendicitis who did not receive an appendectomy and recovered appropriately [9].

Guidelines for mesh use are less definitive, with most studies advising the avoidance of mesh in the presence of infection or contamination [10]. In clean-contaminated cases, tissue repair (such as Desarda) offers a safe and effective alternative, while in select patients at a high risk for recurrence, resorbable or biologic mesh may be considered [11,12]. Reports of staged or delayed mesh repair have also demonstrated success [13,14]. In cases without appendicitis, mesh placement has been well-documented with successful use for hernia repair [15,16]. In one case of a patient with an open Amyand’s hernia repair with appendectomy, synthetic mesh placement was used with no complication two weeks postoperatively [17]. However, synthetic mesh placement also carries the risk of creating a nidus for infection. For this reason, the Losanoff and Basson guidelines do not recommend mesh placement in the case of appendicitis or other infection.

It is important to underscore that individualized assessment - rather than rigid adherence to guidelines - should guide operative decision-making. For example, in our patient with significant structural damage to the inguinal canal, mesh repair was necessary to minimize recurrence risk despite there being a higher risk of infection from the presence of cecal ischemia. Resorbable biosynthetic mesh was utilized to mitigate this risk while adding greater support than a tissue repair only. Decisions between tissue and mesh repair should ideally incorporate both surgical and patient-specific factors.

## Conclusions

Amyand’s hernia remains a rare and diagnostically challenging condition. Our experience supports appendectomy for inflamed appendices and tissue repair in contaminated fields, with consideration of biosynthetic mesh in select patients at high recurrence risk. Continued refinement of management algorithms may further improve outcomes in this uncommon clinical scenario.
